# Intermittent Fasting: Efficacy, Safety, and Its Impact on Body Weight, Glucose Metabolism, and Gut Microbiota

**DOI:** 10.7759/cureus.97773

**Published:** 2025-11-25

**Authors:** Patryk Brasse, Julia Zerdka, Karolina Staszkiewicz, Kacper K Staszkiewicz, Mateusz Piszka, Eliza Kwapien, Jakub Bartkowski, Maria Kubicka, Filip Czarnecki

**Affiliations:** 1 Medicine, Wojewódzki Szpital Specjalistyczny nr 5 im. św. Barbary, Sosnowiec, POL; 2 Orthopedics, SP ZOZ MSWiA, Katowice, POL; 3 Medicine, Miejskie Zakłady Opieki Zdrowotnej w Żorach, Żory, POL; 4 Medicine, Samodzielny Publiczny Szpital Kliniczny im. Andrzeja Mielęckiego w Katowicach, Katowice, POL; 5 Medicine, Szpital Miejski w Siemianowicach Śląskich, Siemianowice Śląskie, POL

**Keywords:** autophagy regulation, glycated hemoglobin (hba1c), gut microbiome, “intermittent fasting”, nutrition and metabolism, obesity and diabetes

## Abstract

Intermittent fasting (IF) has gained significant attention as a nutritional strategy with potential health-promoting effects. It typically involves periodic restriction of calorie intake or limiting the daily eating window. Growing scientific interest has led to a wide range of clinical and observational research exploring its biological and metabolic impacts.

Existing studies suggest that IF may positively influence metabolic health, weight management, and cellular processes. However, the breadth of findings varies, and questions remain regarding its long-term safety and effectiveness across diverse populations, especially individuals with chronic conditions. A comprehensive evaluation of the current evidence is therefore warranted.

An analysis of 55 publications, including clinical trials, observational studies, and review articles, was conducted. These sources were examined to assess the effects of various forms of IF on metabolic health, diabetes-related outcomes, gut microbiome composition, and broader physiological functions.

Across most studies, IF was associated with moderate but clinically meaningful weight loss, enhanced insulin sensitivity, reductions in blood pressure, and improvements in lipid profiles. Some evidence also pointed to beneficial changes in gut microbiota, decreased oxidative stress, and increased autophagy activity. Comparisons with traditional caloric restriction indicate similar levels of effectiveness and safety, with IF potentially offering better adherence for certain individuals. Despite these promising outcomes, the current evidence base lacks long-term studies and comprehensive evaluations in specific clinical populations, limiting conclusions about sustained benefits and generalizability.

IF appears to be a promising approach for the prevention and management of obesity and metabolic disorders. Its combination of physiological benefits and relative ease of implementation makes it an appealing strategy. Nonetheless, further long-term and population-specific research is needed to fully establish its safety, durability, and applicability across diverse groups.

## Introduction and background

Intermittent fasting (IF) has been dynamically gaining popularity in recent years, which may be a response to the growing problem associated with obesity and its complications, such as diabetes, hypertension, or coronary artery disease. Obesity contributes to 2.8 million deaths per year, which is why it is a significant challenge for health care today [1,2]. Against the background of this growing health problem, new and more effective nutritional strategies are being sought. One of them is IF.

Unlike traditional caloric restriction (CR) strategies that focus on what or how much to eat, IF focuses on the timing of food intake. Consequently, IF is defined not as a specific diet composition, but as an umbrella term for various eating patterns that cycle between periods of voluntary fasting and eating. The time of eating meals is strictly limited, without the need for complete CR. Unlike classic weight loss diets, IF focuses on the timing of food intake rather than its composition. To understand the effectiveness of this intervention, it is necessary to understand the underlying physiological mechanism known as the "metabolic switch." During the fasting window, the body shifts from using glucose (derived from recent meals) as its primary fuel source to using fatty acids and ketone bodies derived from stored adipose tissue. This transition is believed to trigger various cellular repair processes and metabolic improvements that are not activated during standard eating patterns. In addition, this way of eating can lead to numerous health benefits resulting from a positive effect on the regulation of the intestinal microbiome, activation of autophagy processes, or weight reduction [3].

The most studied models of IF include three main eating patterns. They differ in the length of the fasting period and the frequency of meals.

Time-restricted eating (TRE) involves limiting your eating to a specific time window during the day, typically between four and 10 hours. The most used variant is the 16:8 model, in which fasting is maintained for 16 hours, and food is allowed only in the remaining eight hours. This approach does not limit the type or number of products consumed, but focuses on their consumption at a certain time, which helps maintain the circadian rhythm and regularity of metabolism.

Alternate-day fasting (ADF) involves alternating fasting days and days of normal eating. On fasting days, energy intake is significantly limited - usually to 20-25% of the daily caloric requirement, which corresponds to one small meal. On other days, dietary restrictions are not applied. This model requires more discipline but provides clear cyclical periods of metabolic rest.

Diet 5:2 consists of using two consecutive fasting days a week, during which the supply of calories is limited, with five days of normal meal intake. This model is considered more flexible and easier to maintain in the long term, making it popular in clinical practice and among healthy individuals.

While weight loss is the most common reason for adopting IF, its impact extends beyond a simple reduction in body mass. Scientific evidence indicates that periodic energy restriction leads to improved insulin sensitivity, lower blood pressure, and reduced risk of metabolic diseases [4,5]. However, the effectiveness of these interventions is not uniform and depends heavily on the specific model used, the duration of adherence, and individual patient characteristics.

A critical, yet often overlooked, component of this metabolic puzzle is the gut microbiome. Emerging research suggests that the composition of intestinal bacteria fluctuates significantly with nutrient availability. The gut microbiome is now hypothesized to be a key mediator in the relationship between IF and metabolic health, influencing host energy harvest and systemic inflammation. Therefore, the purpose of this narrative review is to synthesize current research on not only the metabolic parameters (such as weight and insulin resistance) but specifically how IF remodels the gut microbiome to influence these outcomes.

## Review

Methods

This work is a narrative review of the literature, the aim of which was to collect and systematize current scientific data on the effect of IF on metabolism, physiological functions, risk of metabolic diseases, and the gut microbiome.

The search for publications was carried out in the databases: PubMed, Scopus, and Google Scholar using keywords such as “intermittent fasting”, “time-restricted eating”, “alternate-day fasting”, "metabolic switching", “autophagy”, “microbiome”, “metabolism”, “insulin resistance”, “diabetes”, and “obesity”. The search included works published up to October 2025, with no geographical restrictions. Only peer-reviewed publications available in full text, in English or Polish, were included.

We selected 55 studies for inclusion, which included a wide range of evidence: randomized controlled trials, observational and animal experimental studies, as well as selected systematic and narrative reviews. The primary focus for inclusion was the topic of IF's biological mechanisms (including "metabolic switching" between glucose and fatty acid/ketone body metabolism), its impact on key metabolic parameters (body weight, insulin resistance, lipid profile, blood pressure, glycated hemoglobin), and its effect on gut microbiota composition. Publications such as commentaries, case studies, preliminary reports, and studies deemed to be of low methodological quality were excluded.

The collected material includes 55 literature items; 85.45% (47) of the discussed studies date from 2016 to 2025, confirming the high level of relevance of the literature. The duration of interventions in IF studies varied. It ranged from four weeks to 12 months, with the most common being eight to 12 weeks. The study populations consisted mainly of overweight or obese adults, to a lesser extent, those with prediabetes or type 2 diabetes mellitus, as well as a small group of healthy adults. In most studies, the number of samples ranged from 40 to 150 participants, although the largest studies included even more than 1400 people.

The methodological quality of most studies was rated as moderate to high. This was due to randomization, the presence of control groups, and standardized metabolic measurements (glucose, insulin resistance, lipid profile, body weight, body composition).

Physiology of intermittent fasting

During fasting periods, numerous metabolic and hormonal adaptations take place in the body, which switch the metabolism from the "feeding" phase to the "hunger" phase. This "metabolic switch" is recognized as the main target and mechanism that is achieved in IF, which in turn triggers beneficial physiological changes in the body. In the first hours after a meal, energy comes mainly from glucose (delivered to cells after an intense increase in insulin secretion by the pancreas), and excess carbohydrates are stored as glycogen in the liver and muscles. As the time since the last meal increases, glycogen stores are gradually depleted through glycogenolysis, which allows blood glucose levels to be maintained. This process occurs primarily in the liver, which plays a key role in this phase, which lasts up to about 24 hours of fasting [6,7]. When glycogen stores are significantly depleted, the body activates alternative energy pathways, which form the basis of the physiology of IF.

In a state of starvation, triglycerides are mobilized from adipose tissue. Lipolysis breaks down triglycerides into free fatty acids and glycerol, which enter the bloodstream. In women, the process of non-oxidative oxidation of fatty acids occurs 50% faster than in men [8]. The liver converts some of the free fatty acids into ketone bodies, which become new fuel for many tissues, including the brain, when glucose availability decreases [9]. This "metabolic switch" (from the use of glucose to the use of fats and ketones) is one of the key actions of IF. The "metabolic switch", caused by periods of food restriction, has its natural analogues in nature. Periodic starvation, leading to the "metabolic switch" and the activation of physiological processes, is a common element in the animal world. It enables organisms to survive periods of food shortage and exerts beneficial pleiotropic effects not only by saving energy, but also by strengthening adaptation mechanisms [10,11].

IF has a significant effect on metabolic changes by modulating hormone secretion. In a fasting state, insulin levels decrease, which promotes lipolysis and reduces fat storage. At the same time, the ratio of glucagon to insulin increases, which stimulates gluconeogenesis in the liver and the release of glucose into the blood [12]. In addition, a decrease in insulin-like growth factor (IGF-1) and inhibition of mammalian target of rapamycin (mTOR) signaling are observed, which silences growth and proliferative pathways and activates cell repair mechanisms. In response to a decrease in energy availability, the activity of AMPK (AMP-activated protein kinase) - the energy sensor of cells - is increased, which promotes catabolic processes such as the breakdown of fats and inhibits the synthesis of lipids and proteins [13].

At the level of the nervous system, IF induces beneficial neurochemical changes. Ketone bodies provide fuel for neurons and stimulate neuroprotective pathways, for example, by increasing the activity of brain-derived neurotrophic factor (BDNF), improving synaptic plasticity, and increasing resistance to neuronal stress. This influence may be important in the context of neurodegeneration and delay of brain aging processes [10].

When discussing the health benefits of IF, its effect on inducing the process of autophagy cannot be overlooked. It is a "cellular cleaning" process in which damaged organelles, proteins, or other unnecessary elements are removed. This mechanism allows cellular components to be recycled and energy to be provided during periods of deficiency. In the context of IF, autophagy supports cell cleansing, supports adaptation by eliminating metabolic stress, and can counteract the aging process and the development of metabolic diseases. When fasting every other day for eight weeks (24-hour fasting, three days/week) is used in mice, there is a significant increase in autophagy markers in the liver, including an increase in LC3 protein, an increase in mRNA for Map1lc3b, Beclin-1, and an increase in LAMP1, especially in subjects fed a standard diet, however, no similar activation was observed in skeletal muscle. In humans, on the other hand, mRNA changes were found for some markers of autophagy in muscles (SQSTM1, Beclin-1, LAMP2) after 24-hour fasting, but these results were smaller and associated with the effect of weight loss [14]. This may be due to the fact that a 24-hour fast in mice, because of differences in metabolism and lifespan, does not correspond directly to a 24-hour fast in humans. Therefore, further research on fasting in humans is particularly important.

The study by Hofer et al. found that fasting or calorie restriction (CR) causes a rapid increase in levels of spermidine, a natural compound present in cells. This increase is necessary to start the autophagy process. This mechanism is based on the effect of spermidine on the eukaryotic translation initiation factor 5A (EIF5A), which supports the production of transcription factor EB (TFEB) protein - crucial for the activation of autophagy [15].

An improvement in lipid profile, a decrease in fasting glucose, and a reduction in insulin resistance were observed during early time-restricted eating (eTRE), despite the absence of a significant change in participants' body weight. These results show the beneficial effects on health of fasting itself, regardless of weight reduction. Additionally, study participants had lower blood pressure readings and reduced levels of oxidative stress markers, suggesting an improvement in overall metabolic and cardiovascular health. An important finding was also the reduction of subjective feelings of hunger, despite the shortened eating window, which indicates the potential adaptive benefits of this type of intervention. This study proves that modifying the circadian rhythm of eating regardless of the calorie deficit alone can have a beneficial effect on carbohydrate metabolism and cardiometabolic health. The results suggest that shifting meal intake to earlier in the day may support physiological metabolic rhythms and improve the health profile of people with glucose disorders [16]. Studies have shown that IF can have similar effects to continuous CR [17].

In summary, the physiology of IF includes the transition of metabolism from glucose to fats and ketones, hormonal adaptation with insulin reduction and glucagon activation, activation of AMPK and inhibition of mTOR, enhancement of autophagy and repair mechanisms, modulation of oxidative stress and inflammation, as well as neuroprotective effects. These mechanisms work together, allowing the body to adapt to periods of limited access to energy while supporting long-term metabolic and cellular health.

Which form of intermittent fasting is the most effective?

The effectiveness of IF may depend on the diet regimen adopted. Various forms of IF, such as TRE 16:8, IF 5:2, or ADF, have been described in the literature, which differ in the length of fasting and eating periods. In recent years, studies have appeared directly comparing these approaches, making it possible to assess their effectiveness in reducing body weight and improving metabolic parameters.

A study published in August 2025 compared the effectiveness of two popular IF methods: 5:2 (two days of fasting per week) and 16:8 (a dietary window of 8 hours a day) in overweight and obese people. The results suggest that both methods show similar effectiveness in weight loss [18].

An analysis published in 2024 suggests that all forms of IF have the potential to improve metabolic health; however, fasting every other day (ADF) may produce better results in terms of weight loss, improved lipid profile (except high-density lipoprotein cholesterol (HDL-C)), and insulin sensitivity. Unfortunately, this evidence was characterized by low or even very low evidentiary certainty. Additionally, it should be noted that ADF may be more difficult to maintain in the long term [19].

The study by Derron et al. compared the effectiveness of fasting every other day (ADF) and a restricted eating window (TRE - 16:8) in adults. In a randomized clinical trial, ADF was shown to be more effective than TRE in weight reduction; however, this was mainly associated with a greater energy deficit. The authors suggest that ADF may be a more effective approach in short-term fat reduction, although the study had limitations such as short duration and small sample size [20].

A study released in Frontiers in Nutrition in 2024 conducted a networked comparative analysis of four IF methods in people with type 2 diabetes: twice-weekly fasting, a fasting mimic diet, a restricted eating window (TRE), and IF. The analysis included 13 randomized trials involving 867 patients. All IF strategies showed better effects in controlling glucose levels and insulin sensitivity compared to the standard diet. However, the results of the online comparative analysis did not show significant differences between the individual methods of IF. A rank analysis of the SUCRA indicated that fasting twice a week was the most effective method in improving metabolic parameters. The authors emphasize the need for further studies with a longer follow-up period and larger sample size to confirm these results and evaluate the long-term effects of different IF methods [21].

The effectiveness of IF depends on the method chosen, with all common regimens (16:8, 5:2, ADF) being able to lead to weight loss and improved metabolic parameters. Fasting every other day (ADF) shows the greatest potential in reducing body fat in the cited studies. Differences between methods are generally small, and the evidentiary certainty remains moderate to low, highlighting the need for further long-term research.

Intermittent fasting - effectiveness and safety

The effectiveness of IF in reducing body weight and improving metabolic parameters is the subject of intensive scientific analysis. Different IF regimens show varying efficacy, and comparisons with traditional CR allow you to assess which strategies are most effective and best tolerated by participants. Although IF has many benefits, this diet should also be analyzed for side effects, potential complications, and effectiveness, especially when compared to traditional CR.

A randomized study of 165 participants compared the 4:3 IF method (three days of fasting per week) with daily calorie restriction (DCR). At 12 months, the IF group achieved better weight loss results than the DCR group (mean difference of 2.89 kg (95% CI, 5.65 to 0.14 kg); p = 0.040) [22]. However, there is no shortage of evidence in the scientific literature that IF is not more effective in reducing body weight than traditional CR.

Table 1 presents the results of selected original studies from 2016 to 2025 comparing the effectiveness of IF with traditional CR in weight reduction. Most studies have shown that both strategies lead to comparable weight loss, with some forms of IF, especially ADF (every other day fasting) and the 5:2 method, may slightly increase the rate of weight reduction in the short term. However, these differences rarely achieved statistical significance. The results suggest that both IF and CR are effective methods of weight control, and the choice between them should depend on individual preferences, dietary ability, and patient tolerance [23-26].

**Table 1:** Comparison of diets - intermittent fasting with calorie restriction ADF, alternate-day fasting; CCR, continuous calorie restriction; IF, intermittent fasting; KD, ketogenic diet; MedDiet, Mediterranean diet; mADF, modified alternate-day fasting; TRE, time-restricted eating

Source and year	IF vs. CR protocol	Duration	Participants	Weight loss outcomes	Conclusions
Trepanowski et al., JAMA Internal Medicine (2017) [23]	ADF vs. CCR	12 months	100 people with obesity	ADF -6.0% vs. CR -5.3% body weight	Both methods effective, no significant differences
Schübel et al., The American Journal of Clinical Nutrition (2018) [24]	IF (5:2) vs. CCR	50 weeks	150 people with overweight and obesity	After 50 weeks: IF -5.2% vs. CR -4.9%	Similar effectiveness, slight difference in favor of IF
Catenacci et al., Obesity (2025) [25]	Zero-calorie ADF vs. CCR (-400 kcal/day)	Eight weeks + follow-up	26 adults with obesity	ADF -8.2 kg vs. CR -7.1 kg	No statistical differences, better body composition in IF
Martínez-Montoro et al., BMC Medicine (2025) [26]	mADF, TRE, KD vs. MedDiet (with calorie restriction)	Three months	140 adults	Greater weight loss in mADF, KD and TRE than in the MedDiet	KD proved to be the most effective

More broadly, different forms of IF (TRE, 5:2 fasting, and ADF) appear to be comparable to, or slightly more effective than, traditional CR in reducing body weight and improving metabolic parameters. IF may therefore be an alternative method of weight control and metabolism, but it requires further verification in high-certainty studies, especially in populations with metabolic diseases [19].

IF also offers additional benefits in the context of metabolic liver disease (metabolic dysfunction-associated steatotic liver disease (MAFLD)). A randomized trial of patients with this condition compared a 5:2 diet with DCR. After 12 weeks of following these diets, the two groups did not differ significantly in terms of weight loss, metabolic rates, or lipid profile. It is worth mentioning, however, that the group using 5:2 IF showed a significantly greater reduction in the severity of fatty liver disease and the degree of fibrosis, as well as better results in ultrasound measurements of liver parameters (controlled attenuation parameter (CAP), liver stiffness measurement (LSM)), regardless of weight loss [27]. This study provides further evidence that the IF diet may offer additional benefits regardless of weight reduction - in this case, in the context of liver disease (MAFLD).

In the context of type 2 diabetes, IF remains a strategy with not fully confirmed safety. Existing data indicate that it may lead to improved insulin sensitivity and moderate weight reduction, but the number of high-quality studies is limited [28].

A study published in 2023 analyzes the effects of IF on health and life expectancy, considering both benefits and potential risks. This analysis uses the Integrated Model of Risk and Survival (IMRS) model, which takes into account different risk factors and life expectancy. The results suggest that IF may contribute to improved metabolic health and reduced risk of chronic disease, which may lead to a longer life. However, in the short term (one year), IF may increase the risk of death. This may be due to the body's initial reactions to changes in diet and metabolism [29].

IF should also be analyzed in subjects who are burdened with disease. During Ramadan, a significant increase in the number of deaths was observed among patients undergoing hemodialysis compared to other months, which may have been related to changes in dialysis schedule, dehydration, hypotonia, and increased physical and mental stress during fasting. The authors suggest the need to adjust dialysis schedules and monitor patients' conditions during Ramadan to minimize the risk of complications. This study does not directly address IF in the context of healthy individuals, but it analyzes the effects of religious fasting on patients with chronic kidney failure. However, this study provides us with important information that people burdened with chronic diseases may be particularly vulnerable to complications of fasting, which requires further intensive analysis [30].

In 2019, an observational study was conducted on 1422 participants fasting for four to 21 days. It assessed the impact of fasting on health, well-being, and safety. Side effects were observed among the participants, the most common of which were: headaches, dizziness, constipation, nausea, and mood swings. These symptoms were usually transient and resolved after an adaptation period. They occurred in less than 1% of the subjects. The study suggests that fasting in this time range is safe for healthy people, provided that proper preparation and monitoring of health status are provided, and in the case of existing medical conditions, it is recommended to consult a doctor before starting fasting [31].

Effect of intermittent fasting on obesity and weight reduction

IF has become one of the most studied nutritional strategies in recent years to support weight reduction and improve metabolic parameters. However, the results of clinical trials to date provide mixed conclusions about the effectiveness of this intervention compared to classical CR.

One study in this area is the randomized trial of Trepanowski et al., which compared fasting every other day of ADF (25% of energy requirements on fasting days and 125% on unrestricted days), CR (a steady reduction to 75% of the requirement each day), and a control group. After one year of intervention, there were no significant differences between the ADF and CR groups in terms of weight loss, blood pressure, heart rate, lipid profile, fasting glycaemia, insulin resistance (HOMA-IR), C-reactive protein, or homocysteine levels. The average weight loss was about 6% in both intervention groups; however, IF was less tolerated and had a higher dropout rate among participants. The authors emphasized that despite the popularity of IF, ADF did not provide clear metabolic benefits or weight reduction advantages compared to traditional DCR [23].

Similar conclusions were found in a short-term study by Tempelman et al., which evaluated the impact of 24-hour fasting every other day in thin people. At the same time, it should be emphasized that the sample size was small and the duration of the intervention was limited to three weeks, which significantly limited the statistical power of the study and the possibility of generalizing the results. The analyzed data suggested that in people with normal body weight, the potential metabolic benefits of IF depend mainly on energy deficit rather than meal rhythm alone [32].

Similar conclusions emerge from the work of Sundfør et al., who compared fasting for two days a week with DCR. Both strategies had similar effects in reducing body weight and improving cardiometabolic parameters (blood pressure, triglycerides, and HDL), although IF was less tolerated by participants due to greater hunger [33].

Currently, there is more interest in TRE, which can have a positive effect on metabolism by synchronizing meal consumption with the circadian rhythm. In a study of 90 obese adults participating in a 14-week weight reduction program at the Weight Loss Medicine Clinic at the University of Alabama at Birmingham Hospital, eTRE (7:00 a.m.-3:00 p.m.) combined with CR led to greater weight loss compared to eating meals in a standard 12-hour eating window. Participants using eTRE also achieved significant improvements in diastolic pressure, greater reductions in body fat, and improved mood and overall well-being [34]. These results suggest that the timing of meals, in addition to calorie reduction, may play an important role in improving metabolic outcomes in individuals with obesity.

However, not all forms of TRE show significant effects. A 12-week study comparing TRE 16:8 to the classic rhythm of three meals a day showed that the differences in weight loss were minimal. The lack of significant improvement in metabolic parameters and the observed loss of lean body mass indicate that simply shifting the hours of eating without a calorie deficit does not guarantee long-term and effective reduction [35].

On the other hand, in a direct comparison of different forms of IF, Derron et al. showed that ADF (alternate day fasting and ad libitum eating days) led to the greatest reduction in total and visceral body fat compared to TRE (eating meals in an eight-hour window of 12:00-20:00) and the control diet. Importantly, only ADF resulted in a significant reduction in visceral fat, triiodothyronine levels, non-HDL cholesterol, and a reduction in basal metabolic rate, suggesting stronger metabolic effects of this regimen. However, it is worth noting that with the help of ADF, the participants achieved the largest calorie deficit. The authors emphasized that ADF may induce more pronounced changes in lipid metabolism and energy management than TRE, although it is associated with a larger energy deficit and potentially more difficult to maintain recommendations [20].

Interesting results were produced by the 16-week EARLY study, in which the 5:2 MR fasting strategy (two days of CR per week) proved to be more effective than pharmacological treatment with metformin or empagliflozin in reducing HbA1c and body weight in patients with early type 2 diabetes [36]. These results indicate the potential of IF as a therapeutic tool in the treatment of metabolic disorders, especially in the early stages of the disease. However, it is important to note the limitations of the study, such as the short duration of intervention (16 weeks) and the unknown durability of effects over a longer period.

On the other hand, in long-term studies conducted in everyday conditions, the effectiveness of IF seems to be lower than in short clinical interventions. In a one-year study comparing IF, Mediterranean, and paleo diets, weight reduction was moderate and similar between groups, and the effect on blood pressure and glycated hemoglobin was statistically insignificant. The authors emphasized that despite the popularity of IF, maintaining long-term dietary recommendations was difficult, and metabolic effects were similar to those observed with less restrictive diets. These results suggest that under real-world settings, without intensive dietary support, the effectiveness of IF is limited and largely dependent on the participants' consequences [37].

Overall, the available evidence indicates that IF may be an effective alternative to traditional weight loss diets, especially in the short term. However, effectiveness depends mainly on the total energy deficit and the ability to maintain the recommendations, rather than on a specific fasting regimen. Strategies based on synchronization with the circadian rhythm, such as eTRE, seem to be the most promising metabolically, while more restrictive forms (ADF, 5:2 MR) may generate faster effects at the expense of lower tolerance and durability in clinical practice.

Effect of intermittent fasting on diabetes

IF has attracted a lot of interest in recent years as a strategy to support the treatment and prevention of type 2 diabetes. Potential mechanisms for fasting effectiveness include increasing insulin sensitivity, lowering glycated hemoglobin, and improving glycemic control not only by reducing weight, but also independently of it.

One study that showed such effects was a study by Sutton et al., which evaluated eTRE in men with prediabetes. Compared to the standard 12-hour regimen, it led to a significant reduction in fasting and post-glucose insulin levels, as well as improvements in insulin sensitivity and β-cell function, despite no changes in glycaemia. These results confirm that synchronizing meal consumption with the circadian rhythm can improve carbohydrate metabolism regardless of weight loss [38]. Similar observations were noted in the work of Wegman et al., in which IF was associated with a significant decrease in plasma insulin levels [16].

In turn, in a randomized trial, Obermayer et al. the safety and efficacy of IF in patients with type 2 diabetes treated with insulin were evaluated. In a 12-week intervention involving three non-consecutive fasting days per week, a significant reduction in HbA1c was achieved (-7.3 ± 12.0 mmol/mol vs. 0.1 ± 6.1 mmol/mol in the control group; p = 0.012). A reduction in insulin demand has also been observed, indicating that IF may be an effective and safe adjunct treatment of type 2 diabetes, even in people on insulin therapy [39].

These results are also supported by data from a randomized trial of 99 participants, which compared two time-restricted TRE feeding regimens (16:8 and 14:10) used three times a week for three months. Both variants led to a significant reduction in fasting glucose (by approx. 30 mg/dL on average) and HbA1c (-0.5%). These data clearly indicate that both TRE 16:8 and 14:10, used several days a week, can effectively improve glycemic control in adults [40].

Further evidence was provided by a study in 120 overweight adults with type 2 diabetes mellitus, which evaluated 12 weeks of time-restricted nutrition (a 10-hour window between 8:00 a.m. and 6:00 p.m.). Participants in this group achieved a significantly greater decrease in HbA1c (-1.54% vs. -0.66%; p < 0.001), fasting glycemia, and HOMA-IR and HOMA-β ratios compared to the control group. In addition, a reduction in doses of hypoglycemic drugs and an improvement in quality of life were observed, which indicates the practical clinical value of TRE in the daily treatment of type 2 diabetes [41].

In a longer, 12-month follow-up, Carter et al. compared IF (two days a week at 500-600 kcal) with continuous CR (1200-1500 kcal per day) in 137 adults with type 2 diabetes. Both strategies led to similar improvements in glycemic control (decrease in HbA1c by approx. 0.4-0.5%) and body weight (-6.8 kg vs. -5.0 kg). No differences were found in lipidogram or hypoglycemia rate, confirming the equivalence and safety of both methods. The results indicate that periodic energy restriction is a safe and equally effective alternative to traditional weight loss diets in improving glycemic control in patients with type 2 diabetes [42].

Other conclusions come from a 12-month study by Gabel et al., which compared ADF (alternate day fasting) with DCR in people with insulin resistance. Although weight loss was comparable (-8% vs. -6%), ADF resulted in a much greater reduction in insulin levels and HOMA-IR than the reduction diet (-52% and -53% vs. -14% and -17%, respectively). These results suggest that ADF may be more effective in improving insulin sensitivity than continuous CR, even with a similar weight loss effect [43].

Overall, available data indicate that various forms of IF, both those based on meal restriction (TRF, eTRF) and intermittent energy deficit (ADF, 5:2), lead to improved glycemic control, decreased glycated hemoglobin, and improved insulin sensitivity in people with type 2 diabetes and prediabetes. Although preliminary data on IF are promising in terms of efficacy and safety in adjuvant diabetes treatment, further research is needed on the long-term efficacy and tolerability of this method in a large study group.

Effects of intermittent fasting on the human gut microbiome

Preliminary reports from scientific studies analyzing the effects of IF on the human microbiome are also promising. Despite the limited number of studies on fasting and the mechanisms underlying the relationship between fasting and the microbiome that are still not fully understood, this area represents an interesting and promising direction for further scientific research.

A study by Su et al. showed that one month of fasting associated with Ramadan (a specific form of religious, intermittent dry fasting observed annually by Muslims) caused significant transient changes in the composition of the microbiome in healthy, non-obese participants. The use of fasting was associated with an increase in the number of bacteria from the Lachnospiraceae family [44]. Representatives of this family, due to the production of butyric acid, a bioactive substance important for the growth of intestinal microorganisms and host intestinal epithelial cells, are associated with protection against colorectal cancer in humans and beneficial metabolic effects [45,46]. Lachnospiraceae may mediate the beneficial effects of IF on human health, but further research is needed to support this assumption.

On the other hand, a study analyzing the effect of three-week IF (5:2) on the gut microbiota, conducted among 72 Chinese volunteers, showed enrichment of the microbiome with *Parabacteroides distasonis* and *Bacteroides thetaiotaomicron* [47]. These species have been linked in previous studies to improved metabolic parameters and weight reduction [48,49].

A 2024 randomized controlled trial compared the use of IF with protein pacing (IF-P) and CR on gut microbiome remodeling and metabolic profile. In the IF-P group, a more pronounced effect on increasing the number of specific families and types of microbes, such as Christensenellaceae, Rikenellaceae, and *Marvinbryantia*, associated with beneficial metabolic profiles was observed [50]. As is known from studies, the relative abundance of Christensenellaceae in the human intestine is inversely proportional to the body mass index (BMI) of the host; similarly, Rikenellaceae has been associated with lower visceral fat, and *Marvinbryantia* with a long-term effect on weight loss in obese people [51-53].

IF may also induce beneficial functional and taxonomic shifts in the microbiome, especially by increasing the number of bacteria producing short-chain fatty acids SCFAs, which may improve lipid and glucose metabolism and positively affect insulin sensitivity [54,55].

Unfortunately, the heterogeneity of IF protocols, different methods of microbiome testing, duration of interventions, and population specificity limit the possibility of unambiguous generalization of conclusions. More long-term research is needed to establish causality between IF, microbiome changes, and metabolic effects in humans.

## Conclusions

IF is a promising strategy to aid weight reduction, improve metabolic parameters, and modulate the gut microbiome. The results of the study indicate that IF improves insulin sensitivity, reduces body weight, lowers blood pressure, glycated hemoglobin, affects the lipid profile, and promotes beneficial changes in the intestinal bacterial flora. This diet can suppress inflammatory processes in the body and even - as a result of autophagy processes - prolong life. However, there was no clear advantage of IF over classic CR in long-term weight control. IF is a well-tolerated and safe method, as long as it is used consciously and under the supervision of specialists in the case of people with chronic diseases. Given the above data, it is not surprising that this dietary intervention is growing in popularity, as it may be a response to obesity-related diseases as well as provide additional health benefits. Due to the promising, though often contradictory, scientific reports on the effectiveness of IF, further long-term, multicenter clinical trials in significant research groups are necessary to assess the actual efficacy of IF as a nutritional intervention and to formulate a final consensus for the medical community.
